# Sarecycline Demonstrates Clinical Effectiveness against Staphylococcal Infections and Inflammatory Dermatoses: Evidence for Improving Antibiotic Stewardship in Dermatology

**DOI:** 10.3390/antibiotics11060722

**Published:** 2022-05-27

**Authors:** Ayman Grada, Mahmoud A. Ghannoum, Christopher G. Bunick

**Affiliations:** 1 Grada Dermatology Research, LLC, Chesterbrook, PA 19087, USA; grada@bu.edu; 2 Center of Medical Mycology, Integrated Microbiome Core, Department of Dermatology, Case Western Reserve University, Cleveland, OH 44106, USA; mag3@case.edu; 3 Yale Department of Dermatology, Yale School of Medicine, New Haven, CT 06520, USA

**Keywords:** antimicrobial resistance, cutaneous infection, dermatology skin disease, drug safety, microbiome dysbiosis, tetracycline antibiotics

## Abstract

Tetracycline class antibiotics are widely used for multiple skin diseases, including acne vulgaris, acne rosacea, cutaneous infections, inflammatory dermatoses, and autoimmune blistering disorders. Concerns about antibiotic resistance and protecting the human/host microbiome beg the question whether broad-spectrum tetracyclines such as doxycycline and minocycline should be prescribed at such a high rate by dermatologists when a narrow-spectrum tetracycline derivative, sarecycline, exists. We evaluated the clinical effectiveness of oral sarecycline against cutaneous staphylococcal infections, eyelid stye, and mucous membrane pemphigoid to determine whether sarecycline is a viable option for clinicians to practice improved antibiotic stewardship. We observed significant improvement in staphylococcal infections and inflammatory dermatoses with courses of oral sarecycline as short as 9 days, with no reported adverse events. These clinical findings are consistent with in vitro microbiological data and anti-inflammatory properties of sarecycline. Our data provides a strong rationale for clinicians to use narrow-spectrum sarecycline rather than broad-spectrum tetracyclines as a first-line agent in treating staphylococcal skin infections and inflammatory skin diseases for which tetracyclines are currently commonly employed. Such advancement in the practice paradigm in dermatology will enhance antibiotic stewardship, reduce risk of antibiotic resistance, protect the human microbiome, and provide patients with precision medicine care.

## 1. Introduction

**Figure 1:**
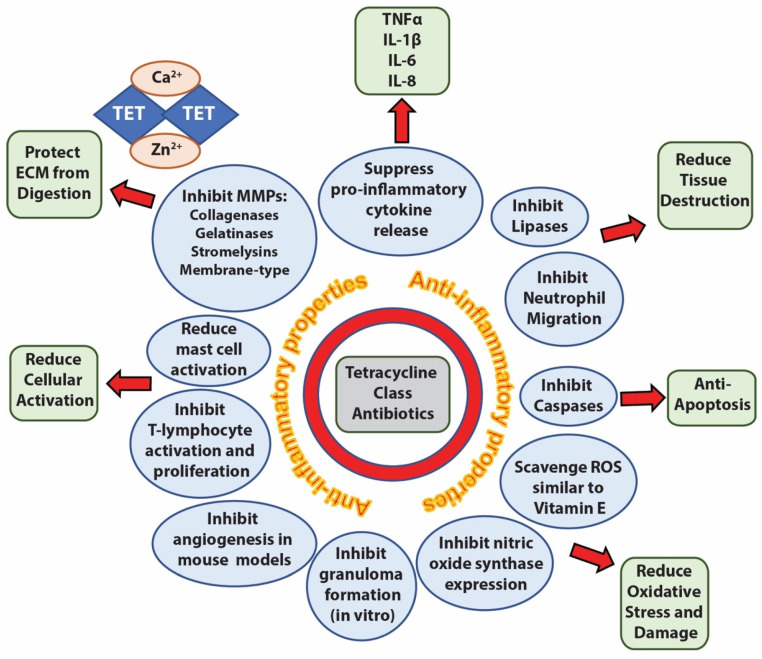
Anti-inflammatory properties of tetracycline class antibiotics. Tetracyclines (TET) can suppress, inhibit, or reduce multiple enzymatic, cellular, and signaling activities (blue circles) leading to protection of tissues from apoptosis, destruction, oxidation, stress, digestion or elevation of inflammatory cytokines (green boxes). One proposed mechanism for TET inhibition of matrix metalloproteinases (MMPs) is through chelation of divalent cations such as calcium (Ca^2+^) and zinc (Zn^2+^).

Dermatologists have the highest antibiotic prescription rate per clinician of any medical specialty [1]. It is estimated that ~68–75% of all antibiotics prescribed by dermatologists are tetracycline class antibiotics, with about two-thirds of those prescriptions being for doxycycline or minocycline [1]. Doxycycline and minocycline are second generation, broad-spectrum tetracycline antibiotics approved by the Food and Drug Administration (FDA) for treatment of numerous Gram-positive and Gram-negative infections, and for “adjunctive use for severe acne vulgaris” [2,3,4]. In addition to their antimicrobial properties, tetracycline class antibiotics are routinely used off-label in dermatology for their purported anti-inflammatory properties, notably inhibition of matrix metalloproteinases, caspases, and lipases, scavenging of reactive oxygen species, reduction in neutrophil migration, and reduction in pro-inflammatory cytokines [5,6,7] (Figure 1).

Widespread use of broad-spectrum tetracyclines has had consequences for patients: emergence of antibiotic resistance among *Cutibacterium acnes* (pathogenic bacterium in acne vulgaris) and other bacterial species, increased perturbation of the gut microbiome leading to gut dysbiosis, and adverse events including gastrointestinal toxicity and vestibular side effects [2,8]. In the United States in 1983, 57% of *C. acnes* isolates from acne vulgaris patients were doxycycline-resistant; this is compared with 79% of *C. acnes* isolates being resistant to clindamycin and 81% resistant to erythromycin [9]. More recent data in the US is lacking, but in 2020, *C. acnes* isolates from acne vulgaris patients in Israel (minocycline 11%, tetracycline 8%, doxycycline 19%, clindamycin 17%, erythromycin 25%) and Jordan (minocycline 3%, tetracycline 36%, doxycycline 37%, clindamycin 56%, and erythromycin 73%) demonstrated substantial antibiotic resistance [10,11]. Similar resistance problems in *C. acnes* were observed in Japan [12]. These antimicrobial resistance (AMR) trends are supported by a systematic analysis on the global burden of AMR in 2019, which estimated 4.95 million worldwide deaths associated with bacterial AMR [13]. Even sub-antimicrobial dosing, a strategy commonly employed by dermatologists to reduce risk of AMR or adverse events during treatment of acne vulgaris or acne rosacea, is increasingly linked to multi-drug resistance [14,15,16,17,18].

The United States Healthcare Infection Control Practices Advisory Committee (HICPAC) and Centers for Disease Control (CDC) published in 2016 an “Antibiotic Stewardship Statement for Antibiotic Guidelines” [19]. The statement recommends physicians “discontinue or narrow unnecessarily broad-spectrum antibiotic therapy” as medically indicated for patients. In dermatology, antibiotic stewardship is emphasized in the 2016 American Academy of Dermatology guidelines for clinical management of acne vulgaris [20]. The continued high rate of prescriptions for broad-spectrum tetracyclines such as doxycycline and minocycline indicates there is room for improvement in antibiotic stewardship among dermatologists [21,22,23].

**Figure 2:**
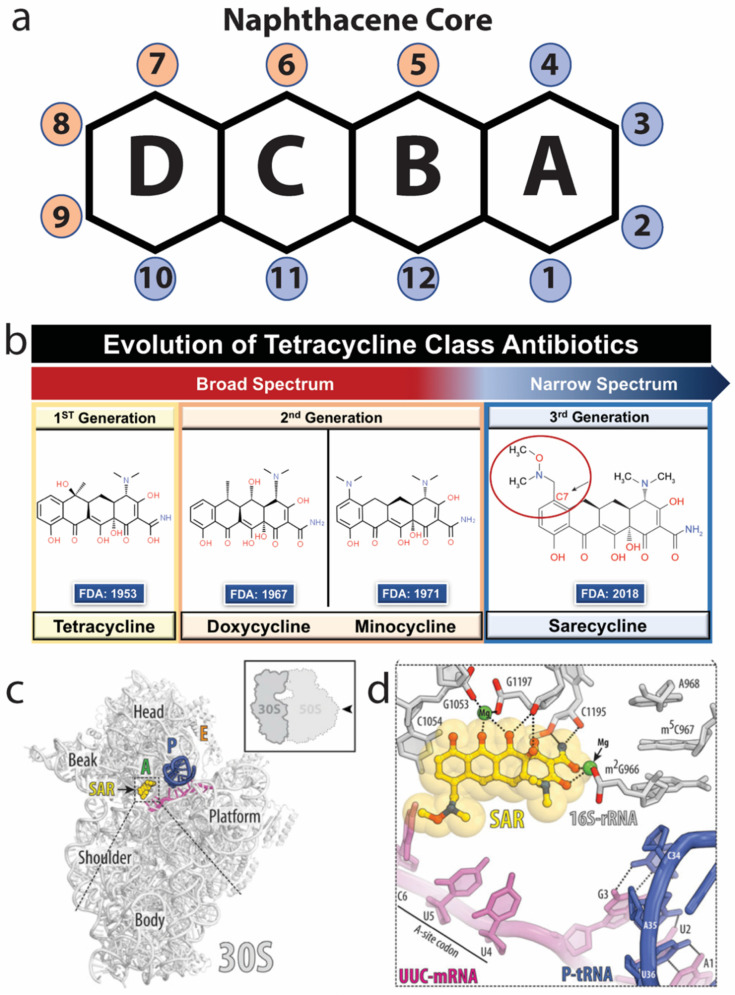
Evolution of chemical and structural properties of tetracyclines over 65 years. (**a**) The four rings of the naphthacene core, the building block of tetracycline antibiotics, are designated “A” through “D”. Carbon positions along the four-ring core are numbered 1–12, with 1–4 and 10–12 colored blue and 5–9 colored orange. The carbon positions colored blue represent the hydrophilic surface of the antibiotic that forms hydrogen bond interactions with the phosphate-oxygen atoms of the 16S rRNA of the bacterial ribosome. The carbon positions colored orange represent the hydrophobic surface that is amenable to chemical modifications without loss of inhibitory activity. Modified from Nguyen 2014 [27] and Batool 2020 [28]. (**b**) Evolution of broad-spectrum tetracyclines over 65 years to the narrow-spectrum sarecycline, which contains a large 7-[(methoxy-(methyl)-amino)-methyl]methyl] group at the C7 position on hydrocarbon ring D (red circle). Modified from Graber 2021 [2]. (**c**) Sarecycline (yellow) is shown bound to the A-site of the decoding center of the 30S *Thermus thermophilus* bacterial ribosomal subunit where it functions to inhibit protein translation. (**d**) Sarecycline (yellow) binds to the 16S-rRNA (gray) of the *T. thermophilus* 30S ribosomal subunit primarily through the hydrophilic face of the molecule, whereas the C7 moiety is able to hydrogen bond and interact with the mRNA (magenta) being transcribed by the ribosome. (**c**,**d**) modified from Batool 2020 [28].

In 2018, the FDA approved a novel, third generation tetracycline-class antibiotic, sarecycline, for clinical treatment of acne vulgaris [24,25,26]. Tetracyclines are named for their common four-ring naphthacene core [27]. Different drugs in the tetracycline class are distinguished by the chemical moiety additions to this four-ring core (Figure 2a) [27]. Sarecycline is unique because it has the longest and largest chemical moiety attached at the carbon-7 (C7) position of ring D of the four-ring core (Figure 2b) [28]. Tetracyclines exert their antibiotic effect by binding to the decoding center of the 30S subunit of the bacterial ribosome, thereby inhibiting bacterial protein translation (Figure 2c). The X-ray crystal structure of sarecycline bound to the 30S subunit of the 70S *Thermus thermophilus* ribosome demonstrated that the sarecycline C7 moiety is involved in molecular interactions with the messenger RNA (mRNA) being translated by the ribosome [28] (Figure 2d).

mRNA interactions in the ribosome have not been observed for other tetracycline class antibiotics, distinguishing sarecycline’s mechanism of action. This biochemical difference is important because sarecycline is also the only tetracycline-class antibiotic with narrow-spectrum activity. Several in vitro and mouse model studies showed sarecycline preferentially works against clinically relevant Gram-positive bacteria over Gram-negative bacteria [29,30,31]. This narrow-spectrum activity is the consequence of a focused design effort to make sarecycline more selective for the Gram-positive bacterium *C. acnes* [32], providing dermatologists a means to improve antibiotic stewardship in acne vulgaris therapy. Against 55 clinical isolates of *C. acnes*, sarecycline had a minimum inhibitory concentration (MIC) ranging from 0.5 to 16 µg/mL [31]. Importantly, the analysis also found sarecycline has MIC_90_ (inhibits 90% of strains tested) of 0.5 µg/mL against 32 isolates of methicillin-sensitive and 31 isolates of methicillin-resistant *Staphylococcus aureus* [31]. This is supported by studies examining sarecycline in murine models of infection [29,31]. First, sarecycline was comparable with doxycycline and minocycline in treating *S. aureus* intraperitoneal infection in mice, but showed reduced efficacy against *Escherichia coli* compared with doxycycline and minocycline, demonstrating its narrow-spectrum effect. Second, sarecycline was as efficacious as doxycycline in treating *S. aureus* in a murine neutropenic thigh model of infection. Lastly, sarecycline was comparable with doxycycline and minocycline in reducing inflammation in a carrageenan-induced rat footpad edema model.

In two large phase III clinical trials treating acne vulgaris, sarecycline demonstrated a favorable safety and tolerability profile compared with broad-spectrum tetracyclines [33]. Narrow-spectrum sarecycline is less active compared with minocycline against 79% of human gut microorganisms tested [30]. Hence, sarecycline “spares the gut microbiome” and reduced activity against Gram-negative gastrointestinal organisms [30,31] accounts for the low gastrointestinal toxicity observed in the phase III clinical trials: treatment-emergent adverse events rates for nausea (~2.1–4.6%), vomiting (1.9−2.1%), and diarrhea (<1%) were low [33,34,35]. The importance of protecting the human intestinal microbiota cannot be overstated, as more evidence emerges about the intersecting roles of a healthy microbiome and overall human health, and the fact that investigations into the long-term impacts of antibiotics on the human intestinal microbiota revealed even short courses of oral antibiotics can have profound effects 2–4 years later [8,36]. Importantly, narrow-spectrum activity does not necessarily mean complete inactivity against Gram-negative organisms; sarecycline can be effective against some Gram-negative bacteria not associated with the human gut microbiota, such as *Empedobacter brevis* [37].

Sarecycline displays additional beneficial safety features: little to no phototoxicity or vestibular disturbance [2], and favorable pharmacokinetics allowing once-daily dosing with or without food [38]. Phototoxicity rates with sarecycline are very low (0.2% to <1%) [33,34], in contrast to a recent pharmacovigilance study suggesting otherwise [39]. The latter study had significant limitations, one of which the authors acknowledged as the “low total amount of adverse events reported with sarecycline” could artificially skew the mathematical calculations of reporting odds ratios to a higher value than in reality. Sarecycline further demonstrated very low (0.4% to <1%) vestibular (dizziness, vertigo, tinnitus) side effects [2,33,34], which is a common problem experienced by patients taking oral minocycline [2]. There are no head-to-head studies comparing different tetracycline class drugs in acne; however, a 52-week long-term extension safety study reinforced that the sarecycline safety profile was consistent with the pivotal trials [34]. Sarecycline is contraindicated in persons who have shown hypersensitivity to any of the tetracyclines [40].

The effectiveness of sarecycline in in vitro and murine studies, its reduced propensity to induce antibiotic resistance, and its established, advantageous safety profile raises the question whether sarecycline has clinical utility beyond acne vulgaris. To further understand the clinical capabilities of sarecycline, we treated three patients with staphylococcal infection and two patients with inflammatory skin disease using oral sarecycline. We describe here these five patients and the clinical improvement observed.

## 2. Results

### 2.1. Patient 1

**Figure 3:**
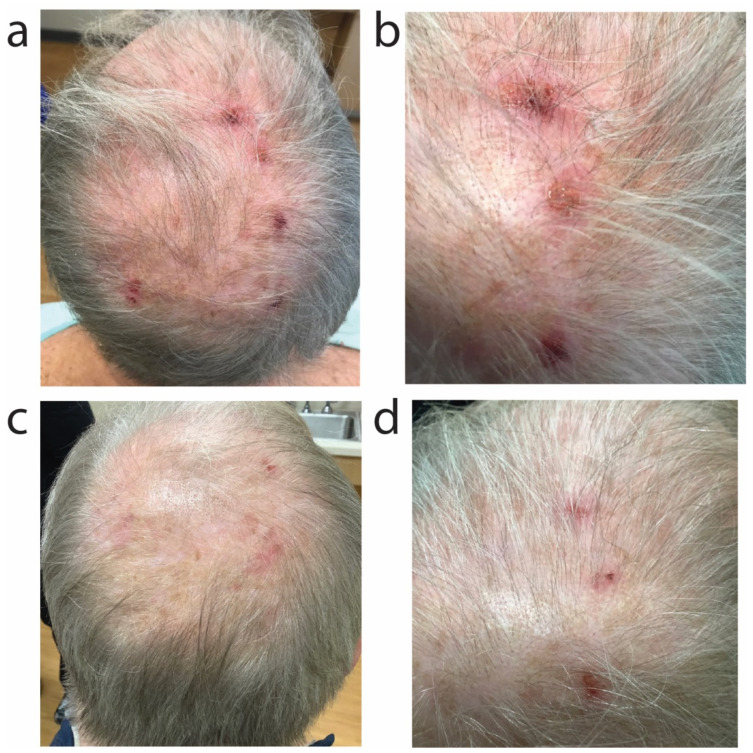
Scalp folliculitis due to methicillin-sensitive *S. aureus* improved with oral sarecycline. Global (**a**) and zoomed-in (**b**) clinical photos of the head prior to initiation of sarecycline. Global (**c**) and zoomed-in (**d**) photos demonstrating clinical improvement of the scalp 11 days later, after the patient completed 9 days of oral sarecycline.

A 67-year-old Caucasian male with history of atopic dermatitis (on dupilumab 300 mg every other week since November 2020), chronic kidney disease, gouty arthritis (on allopurinol and colchicine), nephrolithiasis, Crohn’s disease of the small bowel and colon, and depression (on Lexapro) presented for pruritic, crusted papules on the scalp that he admitted to picking at frequently (Figure 3a,b). Initially, a two-week course of topical clobetasol 0.05% cream twice daily to the scalp did not improve the papules or the itch. Upon return to clinic, a superficial aerobic culture of the scalp papules identified heavy growth of methicillin-sensitive *Staphylococcus aureus*. The patient was initiated on oral sarecycline 100 mg daily for 9 days for treatment of staphylococcal folliculitis of the scalp. He demonstrated significant clinical improvement of the scalp 11 days after sarecycline initiation (Figure 3c,d), and experienced reduction in itch.

### 2.2. Patient 2

**Figure 4:**
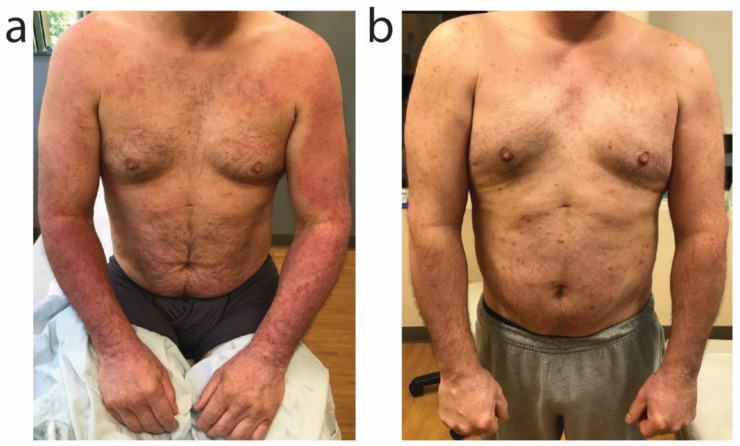
Impetiginized atopic dermatitis improved with oral sarecycline. Impetiginized atopic dermatitis of the bilateral upper extremities moreso than the chest and abdomen (**a**) improved after sarecycline 150 mg daily for 9 days (**b**).

A 52-year-old Caucasian male with history of atopic dermatitis associated with IgE elevation to 2732 kU/L (upper limit of normal 114 kU/L) presented for red, pruritic rash over the bilateral upper extremities, chest, abdomen, and bilateral lower extremities (Figure 4a). A superficial aerobic culture from the left forearm identified moderate growth of methicillin-sensitive *S. aureus*. The patient previously used betamethasone 0.05% cream, clobetasol 0.05% cream, triamcinolone 0.1% cream, and tacrolimus 0.1% ointment for his atopic dermatitis with poor efficacy. For treatment of impetiginized atopic dermatitis, the patient was initiated on oral sarecycline 150 mg daily for 9 days (patient weighed 200 pounds), leading to robust reduction in erythema, crusting, and itch (Figure 4b). For long-term atopic dermatitis control, the patient was started on dupilumab 300 mg every 2 weeks.

### 2.3. Patient 3

**Figure 5:**
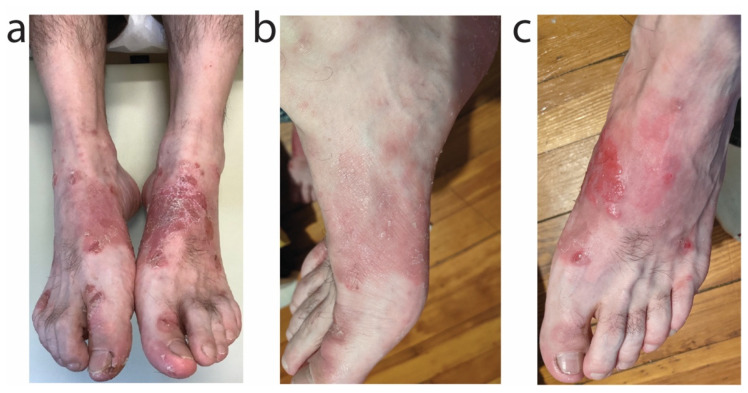
Impetiginized, dyshidrotic atopic dermatitis of the feet improved with oral sarecycline. (**a**) Dyshidrotic atopic dermatitis of the bilateral feet with heavy growth of methicillin-sensitive *S. aureus* on culture. The impetiginized atopic dermatitis of the right (**b**) and left (**c**) feet improved after 9 days of oral sarecycline 100 mg daily.

A 36-year-old Caucasian male with history of atopic dermatitis associated with marked dyshidrosis of the feet moreso than hands and IgE elevation to 465 kU/L (upper limit of normal 114 kU/L) presented with pruritic, erythematous, crusted plaques on the feet despite ongoing therapy with dupilumab 300 mg every 2 weeks (Figure 5a). A superficial aerobic culture from the left dorsal foot identified heavy growth of methicillin-sensitive *S. aureus*. For acute treatment of impetiginized, dyshidrotic atopic dermatitis, the patient was initiated on oral sarecycline 100 mg daily for 9 days, leading to significant reduction in redness, crusting, and itch (Figure 5b,c). For long-term atopic dermatitis control, the patient subsequently was started on twice weekly narrow-band UVB phototherapy of the hands and feet in conjunction with dupilumab 300 mg every 2 weeks and topical clobetasol 0.05% cream twice daily for 2 weeks as needed.

### 2.4. Patient 4

**Figure 6:**
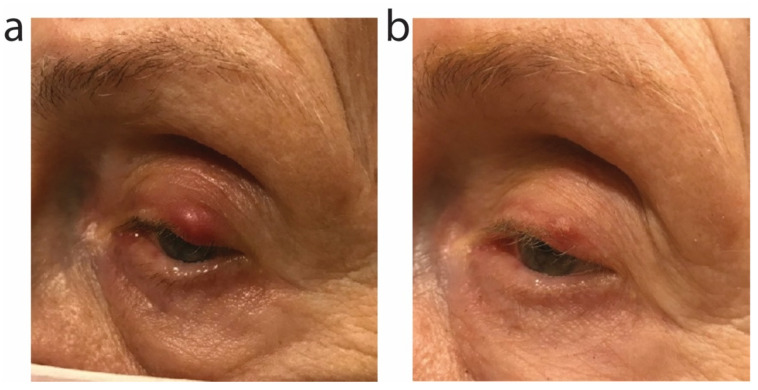
Stye of the left upper eyelid improved with oral sarecycline. (**a**) Clinical photos prior to initiation of sarecycline. (**b**) Clinical improvement of the eyelid stye is observed 15 days later, after the patient completed 9 days of oral sarecycline.

A 83-year-old Caucasian female with history of two basal cell carcinomas of the head presented for routine total body skin examination and complaint of 3 weeks of painful, red lesion on the left upper eyelid that did not improve with warm compresses used 2–3 times daily over those 3 weeks (Figure 6a). The patient was diagnosed with a stye, considered associated with ocular rosacea, and initiated on oral sarecycline 100 mg daily for 9 days. Systemic therapy was chosen over topical therapy because of the pain, duration of lesion, and prior failure of warm compresses. She returned to clinic 15 days later reporting “over 75% reduction” in inflammation of the left upper eyelid (Figure 6b) and complete resolution of pain. She achieved full resolution of the inflammation over the ensuing 3 weeks (not pictured) without further therapy.

### 2.5. Patient 5

A 78-year-old Caucasian female reported influenza infection in January 2019 followed by development of red, blistering, bleeding, and painful lesions in the mouth. Biopsy of the left mandible gingiva in February 2019 revealed “subepithelial vesiculoerosive process consistent with pemphigoid”. The patient was treated with oral clarithromycin 500 mg daily for 8 days with no improvement; additional therapy with niacinamide and probiotics failed. She is a non-smoker with allergy to minocycline and sulfa drugs and medical history of melanoma in situ of the left upper arm and basal cell carcinoma. She was on famotidine at the time of presentation, and this was discontinued because of its association with drug-induced pemphigus [41]. Serum antibodies to BP-180, BP-230, desmoglein-1, and desmoglein-3 were all negative. The patient declined methotrexate and mycophenolate mofetil therapy due to risks of adverse events.

In May 2019, the patient experienced a flare, with 3–5 mm erosions on the left upper gingiva and left buccal mucosa. Mucosal biopsy for direct immunofluorescence (DIF) revealed “linear IgG and C3 and IgA at dermal-epidermal junction, and on roof of salt split skin” consistent with bullous pemphigoid, mucous membrane pemphigoid (MMP), or linear IgA bullous disease. The patient improved with dexamethasone oral rinse. In June 2019 her oral surgeon performed another biopsy which was consistent with MMP, and serum anti-BP180 antibodies were now positive. Since the patient continued to refuse methotrexate (she had family member die while taking methotrexate), dapsone, mycophenolate mofetil, rituximab or intravenous immunoglobulin, she was started on oral doxycycline (with allergist supervision) 100 mg twice daily (BID) in combination with niacinamide 500 mg BID and oral dexamethasone rinse. This combination provided several months relief of her oral erosions, but she complained of severe gastrointestinal upset due to the doxycycline. Upon self-discontinuing the doxycycline and niacinamide, she experienced multiple itchy pink papules on the thighs that on biopsy (H&E and DIF) were consistent with bullous pemphigoid. She was restarted on oral doxycycline 100 mg BID with good control for almost 1 year.

**Figure 7:**
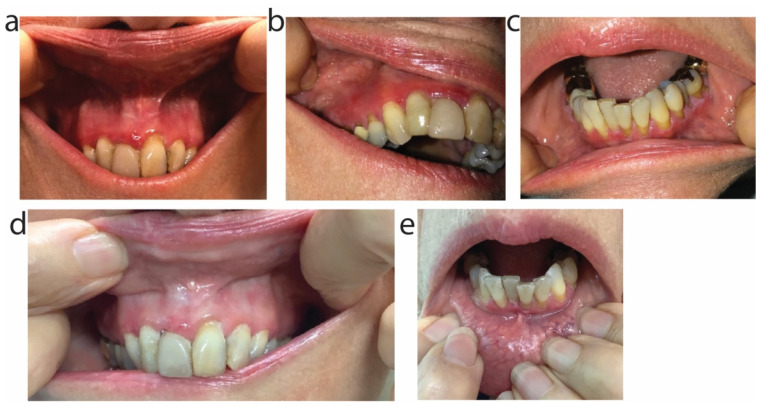
Erosions and inflammation of gingiva due to mucous membrane pemphigoid were reduced and controlled using oral sarecycline. Clinical photos of upper (**a**,**b**) and lower (**c**) gingiva prior to sarecycline use show red erosions along the central and right upper gingiva and erosions along the central lower gingiva. The clinical findings were associated with elevated antibodies to BP180. (**d**,**e**) Clinical improvement of the gingiva was observed 38 days later, after the patient completed 38 days of oral sarecycline 60 mg daily.

In April 2021, the patient presented with new painful erosions in the mouth (Figure 7a–c) and complaints of gastrointestinal distress from doxycycline; it was discontinued and instead the patient started on oral sarecycline 60 mg daily (she weighed 107 pounds) with the thinking that the narrow-spectrum sarecycline would reduce the patient’s gastrointestinal side effects yet maintain clinical efficacy through its anti-inflammatory properties. Over the ensuing month she noted reduced inflammation of the gingiva and no new erosions (Figure 7d,e), along with marked reduction in gastrointestinal upset. The sarecycline dose was increased to 100 mg daily (to try to increase anti-inflammatory effect) and the patient re-started on niacinamide 500 mg once daily. The patient noted she required probiotics in order to tolerate doxycycline, but did not require probiotics while taking sarecycline. The patient was maintained on sarecycline 100 mg daily for 3 months, but due to insurance denying coverage for sarecycline, discontinued the medication in late July 2021, only to experience a flare of her MMP. She restarted doxycycline with recurrence of gastrointestinal upset. In January 2022, after worsening of her MMP and lessening of the COVID-19 pandemic fears, she started mycophenolate mofetil 500 mg BID.

## 3. Discussion

Sarecycline is a novel tetracycline derivative with a bulky C7 moiety that provides it unique chemical properties and mechanism of action distinguishing it from other tetracycline antibiotics [28]. Its narrow-spectrum activity predominantly targets Gram-positive bacteria, notably *C. acnes*, while sparing the human gut microbiome [29,30,31]. Sarecycline is FDA-approved for treating acne vulgaris from age 9 and above [40], and has demonstrated in clinical trials efficacy against facial and truncal acne, often with improvement as early as 3 weeks [25,33,34,42,43]. Beyond acne vulgaris, sarecycline has demonstrated efficacy in treating periorificial dermatitis [44] and papulopustular rosacea [45]. In dermatology, off-label broad-spectrum tetracycline antibiotic use occurs regularly and oral tetracyclines are recommended first-line in treating certain inflammatory and autoimmune disorders: for example, hidradenitis suppurativa [46,47], bullous pemphigoid [48,49,50,51], and mucous membrane pemphigoid [52]. Additionally, doxycycline is the preferred systemic treatment for ocular rosacea [53,54], which commonly manifests with styes and chalazia [55,56].

In the five patients presented here, sarecycline was specifically chosen for its anti-staphylococcal effect, anti-inflammatory properties, and safety profile. The first three patients all had methicillin-sensitive *S. aureus*, and treatment with sarecycline 100 to 150 mg daily for 9 days significantly reduced signs of infection, including crusting and oozing. These patients also experienced marked reduction in redness and inflammation of their skin, which was most noticeable in patient 2 with atopic dermatitis, but also in patients 1 and 3 who had atopic dermatitis flares while on dupilumab therapy. Therefore, the clinical improvement in humans correlated with the anti-staphylococcal data from in vitro and murine studies [29,31]. It is likely, however, that longer courses of sarecycline could have improved the erythema more. Ultimately, sarecycline appears to be a viable, safe medication for dermatologists to use against common clinical problems such as staphylococcal folliculitis, impetigo, and impetiginized inflammatory dermatoses.

Three patients were age 67 or older, and a major consideration in choosing sarecycline in these patients was safety—lowering their risk for gastrointestinal upset, vestibular disturbance, and AMR. This rationale was especially evident in patient 5 with MMP, who experienced gastrointestinal distress with doxycycline, but not during her sarecycline course. The patient’s subjective observation that she required probiotics to endure doxycycline therapy, but not sarecycline, is noteworthy and exemplifies how narrow-spectrum antibiotics can improve the quality of life of patients. For patient 4 with the ocular-rosacea-associated stye non-responsive to warm compresses, the goal was to use a systemic agent to quickly improve her quality of life by reducing her pain. Sarecycline accomplished pain elimination after 9 days of use, however, 9 days was not sufficient to completely eliminate the erythema/inflammation. Perhaps an additional 5–10 days of sarecycline would have achieved this.

None of our patients treated with sarecycline experienced vestibular disturbance, in line with the low 0.4% to <1% observed rate [33,34]. There is a real-world application for this safety feature of sarecycline—use for United States military personnel. It has been noted that minocycline poses risks to United States military personnel because of its high rates of vestibular side effects, leading to restricted use of minocycline for aviators and aircrew members [57]. Sun exposure leading to skin cancer is another issue for military personnel, as experienced by many dermatologists treating our veterans for skin cancer years after their service is complete. With its low vestibular disturbance and low phototoxicity, sarecycline should be considered in United States military guidelines as an excellent choice for treating acne vulgaris and other dermatologic diseases in our servicemen and servicewomen.

Sarecycline is the only FDA-approved oral antibiotic for acne vulgaris that has an FDA-approved label claim for low propensity for development of AMR [40]. The spontaneous mutation frequency for sarecycline is ~10^−10^ at 4–8 MIC (μg/mL) [31]. Having low propensity to induce AMR is a critical characteristic for an antibiotic if it is to be used clinically and promote antibiotic stewardship. Research into the structural mechanism of action of sarecycline identified that the C7 moiety of sarecycline prevents the function of ribosomal resistance proteins, such as TetM, through steric hindrance [28]. This accounts for at least one mechanism by which sarecycline combats AMR.

As dermatology and other medical specialties move further into the practice of precision medicine, “precision” must include the appropriate use of targeted oral antibiotics in order to achieve antibiotic stewardship. This study demonstrates that sarecycline, a narrow-spectrum tetracycline derivative, is clinically effective for patients in the treatment of cutaneous staphylococcal infections, inflammatory dermatoses, and autoimmune blistering disorders. None of the patients in this study reported adverse events to sarecycline. This is important because as dermatologists treat inflammatory skin diseases and autoimmune bullous disorders with oral tetracyclines, they often do so for long periods of time (many months, possibly years). The study is limited by the small sample size, but the results warrant further clinical investigation into the effectiveness of sarecycline across multiple skin diseases, particularly those already being treated by dermatologists with broad-spectrum tetracyclines (doxycycline and minocycline). Sarecycline dosage, frequency of administration, and length of administration are all variables unstudied in skin diseases outside of acne vulgaris.

## 4. Conclusions

Dermatologists can practice precision medicine and advance antibiotic stewardship by utilizing narrow-spectrum antibiotics such as sarecycline rather than broad-spectrum antibiotics. The low propensity of bacterial resistance development to sarecycline, its established safety, and its potential to spare the human gut microbiome necessitate a paradigm shift in how dermatologists prescribe tetracycline-class antibiotics. Although doxycycline and minocycline have broad indications covering Gram-positive and Gram-negative bacteria, their prolonged and intermittent use in dermatologic conditions is not logical given that most cutaneous infections are Gram-positive infections (such as cellulitis, MSSA/MRSA, bacterial folliculitis, etc.) where there is no need for an antibiotic with broad-spectrum activity. The clinical outcomes presented here complement the previously reported acne vulgaris literature, and provide strong rationale for physicians to use sarecycline rather than broad-spectrum tetracyclines as a first-line agent in treating staphylococcal skin and soft tissue infections and inflammatory and autoimmune blistering skin diseases for which tetracyclines are currently commonly employed.

## Data Availability

Not applicable.
